# Supplementary material for: The Systems Biology Research Tool: evolvable open-source software
Source: BMC Syst Biol. 2008 Jun 29;2:55. doi: 10.1186/1752-0509-2-55 (PMC2446383; doi:10.1186/1752-0509-2-55)
Supplement: Additional file 1 — SBRT Archive. An archive of the current version of the Systems Biology Research Tool. [file 1752-0509-2-55-S1.zip › sbrt-1.4.0/doc/developers_guide/api/sbrt/shell/io/package-tree.html]

sbrt.shell.io Class Hierarchy


|  |  |  |
| --- | --- | --- |
| **Overview** \| **Package** \| Class \| Use \| **Tree** \| **Deprecated** \| **Index** \| **Help** | |  |
| **PREV**   **NEXT** | **FRAMES**    **NO FRAMES**     **All Classes** |  |


---


## Hierarchy For Package sbrt.shell.io

**Package Hierarchies:**: All Packages

---

## Class Hierarchy

- java.lang.Object
  - sbrt.shell.io.**AbstractMapInputFile**<K,V> (implements sbrt.shell.io.MapTextInputFile<K,V>)
    - sbrt.shell.io.**ComiFile**<K,V>- sbrt.shell.io.**RomiFile**<K,V>
        - sbrt.shell.io.**ProcessInputFile** (implements sbrt.shell.mng.ManagerInput<K,V>)
    - sbrt.shell.io.**AbstractMapOutputFile**<K,V> (implements sbrt.shell.io.MapTextOutputFile<K,V>)
      - sbrt.shell.io.**ComoFile**<K,V>- sbrt.shell.io.**RomoFile**<K,V>
      - sbrt.shell.io.**AbstractSbrtFile**- sbrt.shell.io.**ComiFileSource**<K,V> (implements java.util.Iterator<E>)- sbrt.shell.io.**ComoFileSource**<K,V> (implements java.util.Iterator<E>)- sbrt.shell.io.**EndOfFile** (implements java.io.Serializable)- sbrt.shell.io.**FileFormat**- sbrt.shell.io.**FileLineIterator**<E> (implements java.util.Iterator<E>)
                  - sbrt.shell.io.**SerializedFileIterator**<E>- sbrt.shell.io.**TextFileLineIterator**<E>
                  - sbrt.shell.io.**MultiOutputFiles**<F,R> (implements sbrt.kernel.io.ResultsManager<T>)- java.io.OutputStream (implements java.io.Closeable, java.io.Flushable)
                      - java.io.FilterOutputStream
                        - java.io.PrintStream (implements java.lang.Appendable, java.io.Closeable)
                          - sbrt.shell.io.**DevNull**
                      - sbrt.shell.io.**RomiFileSource**<K,V> (implements java.util.Iterator<E>)- sbrt.shell.io.**RomoFileSource**<K,V> (implements java.util.Iterator<E>)- sbrt.shell.io.**SerializedInputFile**<E> (implements sbrt.shell.io.InputFile<E>)
                            - sbrt.shell.io.**SerializedMapInputFile**<K,V> (implements sbrt.shell.io.MapInputFile<K,V>)
                            - sbrt.shell.io.**SerializedOutputFile**<E> (implements sbrt.shell.io.OutputFile<T>)
                              - sbrt.shell.io.**SmoFile**<K,V> (implements sbrt.shell.io.MapOutputFile<K,V>)
                              - sbrt.shell.io.**SmoFileSource**<K,V> (implements java.util.Iterator<E>)- sbrt.shell.io.**TextInputFile**<E> (implements sbrt.shell.io.InputFile<E>)- sbrt.shell.io.**TextOutputFile**<T> (implements sbrt.shell.io.OutputFile<T>)- sbrt.shell.io.**WorkingDirectory**- sbrt.shell.io.**XmlFileFactory**

## Interface Hierarchy

- java.lang.Iterable<T>
  - sbrt.shell.io.**InputFile**<E> (also extends sbrt.shell.io.SbrtFile)
    - sbrt.shell.io.**MapInputFile**<K,V>
      - sbrt.shell.io.**MapTextInputFile**<K,V> (also extends sbrt.shell.io.MapTextFile<K,V>)
  - sbrt.kernel.io.**ResultsManager**<T>
    - sbrt.shell.io.**OutputFile**<T> (also extends sbrt.shell.io.SbrtFile)
      - sbrt.shell.io.**MapOutputFile**<K,V>
        - sbrt.shell.io.**MapTextOutputFile**<K,V> (also extends sbrt.shell.io.MapTextFile<K,V>)
    - sbrt.shell.io.**SbrtFile**
      - sbrt.shell.io.**InputFile**<E> (also extends java.lang.Iterable<T>)
        - sbrt.shell.io.**MapInputFile**<K,V>
          - sbrt.shell.io.**MapTextInputFile**<K,V> (also extends sbrt.shell.io.MapTextFile<K,V>)
        - sbrt.shell.io.**MapTextFile**<K,V>
          - sbrt.shell.io.**MapTextInputFile**<K,V> (also extends sbrt.shell.io.MapInputFile<K,V>)- sbrt.shell.io.**MapTextOutputFile**<K,V> (also extends sbrt.shell.io.MapOutputFile<K,V>)
          - sbrt.shell.io.**OutputFile**<T> (also extends sbrt.kernel.io.ResultsManager<T>)
            - sbrt.shell.io.**MapOutputFile**<K,V>
              - sbrt.shell.io.**MapTextOutputFile**<K,V> (also extends sbrt.shell.io.MapTextFile<K,V>)

---


|  |  |  |
| --- | --- | --- |
| **Overview** \| **Package** \| Class \| Use \| **Tree** \| **Deprecated** \| **Index** \| **Help** | |  |
| **PREV**   **NEXT** | **FRAMES**    **NO FRAMES**     **All Classes** |  |


---
